# A Comparison of Ukrainian Hospital Services and Functions Before and During the Russia-Ukraine War

**DOI:** 10.1001/jamahealthforum.2024.0901

**Published:** 2024-05-17

**Authors:** Ubydul Haque, Moeen Hamid Bukhari, Nancy Fiedler, Shanshan Wang, Oleksii Korzh, Juan Espinoza, Miraj Ahmad, Irina Holovanova, Tetyana Chumachenko, Olga Marchak, Dmytro Chumachenko, Osman Ulvi, Iftikhar U. Sikder, Hanna Hubenko, Emily S. Barrett

**Affiliations:** 1 Rutgers Global Health Institute, New Brunswick, New Jersey; 2 Department of Biostatistics and Epidemiology, School of Public Health, Rutgers University, Piscataway, New Jersey; 3 Department of Statistics, Quaid-i-Azam University, Islamabad, Pakistan; 4 Department of Environmental and Occupational Health and Justice, School of Public Health, Rutgers University, Piscataway, New Jersey; 5 Department of Population & Community Health, School of Public Health, University of North Texas Health Science Center, Fort Worth; 6 Department of General Practice—Family Medicine, Kharkiv National Medical University, Kharkiv, Ukraine; 7 Stanley Manne Children’s Research Institute, Ann & Robert H. Lurie Children’s Hospital of Chicago, Chicago, Illinois; 8 Cell Biology & Neuroscience, School of Arts and Sciences, Rutgers University, Piscataway, New Jersey; 9 Poltava State Medical University, Poltava, Ukraine; 10 Epidemiology Department, Kharkiv National Medical University, Kharkiv, Ukraine; 11 Overseas Council–United World Mission, Rivne, Ukraine; 12 Mathematical Modelling and Artificial Intelligence Department, National Aerospace University, Kharkiv Aviation Institute, Kharkiv, Ukraine; 13 Genesis Health System, Davenport, Iowa; 14 Department of Information System, Cleveland State University, Cleveland, Ohio; 15 Department of Public Health, Sumy State University, Sumy, Ukraine

## Abstract

**Importance:**

Since the full-scale Russian invasion, hospitals in Ukraine have been compelled to close or operate at reduced capacity due to inadequate supplies, damage, or destruction caused by war.

**Objective:**

To analyze hospital services in Ukraine during the period before and after the Russian invasion.

**Design, Setting, and Participants:**

Of the 450 hospitals currently functioning in Ukraine, a cross-sectional survey was carried out with the participation of 74 hospitals from 12 oblasts. Hospital administrators responded to an online survey with questions on the use of hospital services. Data were abstracted from hospital databases for the prewar period (before February 23, 2022) and during the war (February 23, 2022, to May 30, 2023).

**Main Outcomes and Measures:**

Hospital services (including emergency services, preventive services, screenings, laboratory tests, obstetrics, telehealth, pharmacy, and rehabilitation services) were compared during the prewar and war periods.

**Results:**

Of 450 Ukrainian hospitals in operation, 74 hospitals (16.0%) across 12 oblasts provided data for the current analyses. During the war, daily emergency admissions increased to 2830, compared with 2773 before the war. At the same time, hospitals reported reduced laboratory testing (72 [97%] vs 63 [85%]), tobacco education (52 [70%] vs 36 [49%]), cancer screening (49 [66%] vs 37 [50%]), gynecological services (43 [58%] vs 32 [43%]), rehabilitation services (37 [50%] vs 27 [36%]), pharmacy services (36 [49%] vs 27 [36%]), and telehealth programs (33 [45%] vs 21 [28%]). Hospitals reported additional difficulties during the war, including disruptions in the supply chain for essential equipment and pharmaceuticals, shortages of laboratory test kits, delays in the delivery of crucial medications, and problems around appropriate medication storage due to power outages.

**Conclusions and Relevance:**

The ongoing war has inflicted profound devastation on Ukraine’s hospitals. The findings of this cross-sectional survey offer valuable insights into the formidable challenges that hospitals confront in war-affected regions and underscore the pressing necessity for bolstering support to sustain and enhance hospital services during wartime.

## Key Points

**Question:**

How has the Russia-Ukraine war affected Ukrainian hospital services and functions?

**Findings:**

In this cross-sectional survey with 74 participating hospitals from 12 oblasts, hospital services were compared during the prewar and war periods. During the war, services related to emergency medical care increased, while other hospital services were notably reduced.

**Meaning:**

These findings offer insights into the formidable challenges that hospitals confront in war-affected regions and underscore the pressing necessity for bolstering support to sustain and enhance hospital services during wartime.

## Teaser

This cross-sectional survey analyzes hospital services in Ukraine during the period before and after the Russian invasion.

## Introduction

War inevitably brings enormous damage and disruption to health care systems.^1,2,3,4,5,6,7^ Before the war, Ukraine had 3 tiers of hospital system: primary hospital units, which include health posts, health centers, and primary hospitals; secondary care provided by general hospitals; and tertiary care served by specialized referral hospitals. Once part of the Soviet Union, Ukraine’s hospital system was deeply rooted in the Soviet regime when the country gained independence in 1991.^8^ Before the full-scale Russian invasion, approximately 720 hospitals were operating in Ukraine. By April 2023, only 450 Ukrainian hospitals were operational. Even before the invasion, the hospital system in Ukraine was already struggling with a shortage of health professionals, inadequate funding, and outdated infrastructure resulting in strains on the provisioning of essential services.^9^ Since the full-scale Russian invasion in February of 2022, these issues have been exacerbated as Ukrainian hospitals grapple with an unprecedented influx of patients (including millions of injured and displaced peoples), the loss of health professionals, shortages of medical supplies, and the destruction of infrastructure, including hospital facilities.^10^ According to the Ministry of Health, Ukraine, from February 2022 to August 2023, medical facilities were damaged and ruined, including health care assets such as hospital transport, personnel, patients, supplies, and warehouses.^10^ As of December 2023, hospitals, clinics, and medical facilities had been attacked and damaged an estimated 661 times with additional assaults on other health facilities such as pharmacies, blood centers, dental clinics, and research institutions.^11,12^

In addition to direct attacks on hospitals, access to medical services has been indirectly affected by security concerns, restricted mobility, broken supply chains, shortages of diagnosis kits and medicine, mass population displacement, and power outages.^13^ The loss of trained personnel has exacerbated this crisis; more than 1% of medical personnel left the country during the war and more than 100 health professionals have been killed as a result of a direct Russian attack. The cumulative outcome of these challenges has resulted in the Ukrainian hospital system being stretched to its limits, struggling to meet the needs of a population in crisis.

It is vital to compare hospital services during the prewar period and the ongoing Russian invasions, in terms of factors such as hospital personnel, service hours and offerings, and storage capacity for vital supplies (eg, blood, vaccines). To that end, this study investigated the type of services provided, losses and challenges, and adaptation and mitigation strategies applied by hospitals since the full-scale Russian invasion began in February 2022.

## Methods

### Survey Participants

Working closely with Ukrainian collaborators in this cross-sectional survey from May 2023 to June 2023, we reached out to administrators at every hospital in Ukraine via email, phone call, and/or text message, requesting their participation in an online survey on the provision of health services before and during the Russian war. A pretested PDF version of the questionnaire in the Ukrainian language was sent to each hospital in advance. Once a hospital agreed to participate, a survey hyperlink was sent for the representatives to complete the form. Data were provided by hospital statisticians from their electronic data systems about the health services before the war (March 2021 to February 2022) and during the war (March 2022 to May 2023). During data cleaning, we cross-checked each variable for accuracy. In total, 74 hospitals provided usable data for analysis.

The survey included 94 questions covering 4 domains: hospital characteristics, key performance indicators, adaptation and mitigation, and cold weather outcomes. Details about the participating hospitals are available in eAppendix in Supplement 1. Informed consent was obtained from the hospital representatives who completed the form. The study protocol was reviewed and approved by the ethical approval committee from Poltava State Medical University in Ukraine and Rutgers University.

#### Hospital Characteristics

Respondents provided basic hospital information (ie, name, location, ownership of the hospital, hospital level [national, oblast, city, or district], and type of hospital [regional, psychiatric, primary and preventive, secondary, tertiary, palliative care, or emergency medical care system and disaster medicine]). We also collected data on the hospital’s location and setting, organizational information, hospital services before and during the war, the loss of hospital professionals (eg, physicians, nurses, lab technicians, and operators), as well as services provided by each hospital, including as emergency and surgery departments, obstetrics and gynecology, auxiliary departments, education, mental health supportive services, and oral health. Respondents were asked to report on the availability of resources (eg, hospital beds, intensive care beds, ambulances/medical transport vehicles, ventilators, etc) and services offered in 2021, before the Russian invasion of Ukraine, as well as during the occupation (February 24, 2022, to May 30, 2023).

#### Key Performance Indicators

A hospital key performance indicator is a performance measure that is used to observe, analyze, optimize, and transform a hospital process to increase satisfaction for both patients and hospitals alike. These metrics are commonly used by care facilities to compare their performance with other care facilities and identify areas of improvement.^14,15^ Specifically, we collected detailed data on hospital operations, services, and staffing. Information was collected from 2 time periods: prewar (March 2021 to February 2022) and during the war (March 2022 to May 2023).

#### Challenges, Adaptation, and Mitigation

In this section, we asked questions regarding the losses and challenges of each hospital, as well as the adaptation and mitigation strategies (new policies implemented to function normally) applied by the hospital since the Russian Invasion of Ukraine (February 24, 2022).

#### Cold Winter Outcomes and Attacks on Health Facilities

Hospitals were asked about the outcomes of power outages and winter on the storage and use of medical products. Location-specific attacks on health facilities were extracted from insecurity insight monitors in Ukraine.^12^

### Statistical Analysis

We first calculated descriptive statistics including mean (SD) for continuous measures and counts and percentages for categorical data. χ^2^ and Fisher exact tests were used to test differences between health care facilities due to small cell counts. Paired sample *t* tests were used for normally distributed continuous variables. Nonparametric tests such as the Wilcoxon signed rank test were applied to variables that were not normally distributed. Further, Cohen *d* effect size was evaluated for each covariate. Repeated measures analysis of variance was used to analyze the changes over time (prewar vs wartime) in hospital-related facilities. Before analysis, multicollinearity was assessed for each covariate (tolerance statistic < 0.4). No evidence of multicollinearity was observed so all variables were retained. Statistical significance was defined based on a 2-sided standard alpha level of .05. R statistical software, version 4.3.1 (R Project for Statistical Computing), was used to perform all the statistical analysis.

## Results

### Hospital Characteristics

Of 450 Ukrainian hospitals in operation, 74 hospitals (16.0%) across 12 oblasts provided data for the current analyses. Of those 74 hospitals, 39 (52.7%) provided secondary health care, 18 (24.3%) primary, 16 (21.6%) tertiary, and 1 (1.4%) palliative health care. Of those, 7 were national-level, 16 were oblast-level, 12 were district-level, and 38 were city hospitals (eTable 1 in Supplement 1). Overall, 60 of the 74 hospitals (81.0%) were communal (ie, owned and operated by local communities or municipalities), while 8 (10.8%) and 6 (8.1%) were state and private hospitals, respectively. A total of 41 participating hospitals (55.4%) were in Kharkiv; 11 (14.9%) were in Kyiv; 9 were in (12.2%) Poltava; and 5 (6.8%) were in Sumy oblasts (eTable 1 in Supplement 1).

### Key Performance Indicators

The use of health services in Ukrainian hospitals changed substantially from the prewar period to after the invasion. From before to after the Russian invasion, the percentage of hospitals offering various services declined across most categories (eTable 2 in Supplement 1). Amidst the war, crucial services were reduced, including laboratory testing (72 [97%] vs 63 [85%]), tobacco education (52 [70%] vs 36 [49%]), cancer screening (49 [66%] vs 37 [50%]), gynecological services (43 [58%] vs 32 [43%]), rehabilitation services (37 [50%] vs 27 [36%]), pharmacy services (36 [49%] vs 27 [36%]) and telehealth programs (33 [45%] vs 21 [28%]) (eTable 2 in Supplement 1). The least affected services include imaging and radiology centers (55 [74%] vs 51 [69%]), routine pediatric services (38 [51%] vs 33 [45%]), intensive care units (ICUs) (44 [59%] vs 42 [57%]), dialysis centers (5 [7%] vs 5 [7%]), addiction treatment centers (5 [7%] vs 5 [7%]), and obstetric deliveries (11 [15%] vs 11 [15%]) (eTable 2 in Supplement 1).

Maternal and newborn health metrics changed substantially with the Russian invasion. In the year before the war, participating hospitals reported a total of 8114 obstetric deliveries compared with 3995 deliveries during the wartime (Table). Of the 3995 wartime deliveries, preeclampsia occurred in 217 (5.4%), preterm birth in 485 (12.1%), and low birth weight in 382 (9.6%). Low birth weight and premature births significantly increased during the war. Furthermore, newborn complications, maternal deaths, labor dystocia, stillbirths, and gestational diabetes declined during the war (Table).

**Table.** Maternal and Newborn Health Metrics Before and During the War in Ukraine

Complication	No. of hospitals reporting birth data	Before war	During war	Statistical test	
		Births with complication, No. (%)	Births with complication, No. (%)	Paired sample *t* test	Cohen *d* (95% CI)
Obstetric deliveries	11	8114	3995	0.041	0.93 (0.04 to 1.81)
Preterm labor	10	1227 (15.1)	484 (12.1)	0.014	1.78 (0.81 to 2.73)
Preeclampsia	10	420 (5.2)	217 (5.4)	0.12	0.72 (−0.19 to 1.63)
Gestational diabetes	10	432 (5.3)	53 (1.3)	0.0006	1.84 (0.77 to 2.89)
Stillbirth	10	397 (4.9)	53 (1.3)	0.0089	1.31 (0.32 to 2.27)
Labor dystocia	10	227 (2.8)	71 (1.8)	0.02^a^	1.14 (0.18 to 2.08)
Maternal death	10	7 (0.1)	1 (0.03)	0.33^a^	0.09 (−0.15 to 0.31)
Newborn complication	7	217 (2.7)	106 (2.7)	0.11	0.93 (0.09 to 1.75)
Low birth weight	10	513 (6.3)	382 (9.6)	0.041	0.38 (−0.51 to 1.26)
Low blood glucose	9	17 (0.2)	22 (0.6)	0.36^a^	0.43 (−0.50 to 1.37)
Premature birth	9	583 (7.2)	485 (12.1)	0.037	0.44 (−0.51 to 1.37)

^a^ Wilcoxon signed-rank test.

### Challenges, Adaptation, and Mitigation

**Figure 1.**
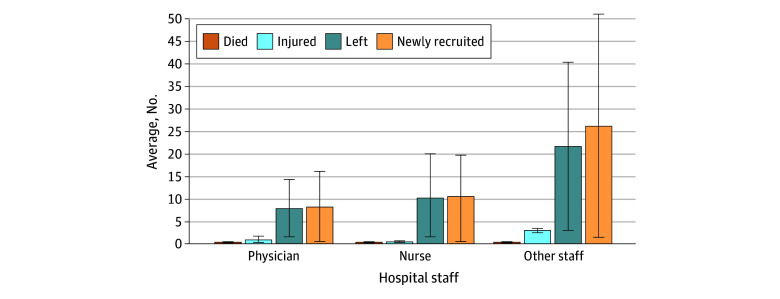
Hospital Staff Losses and Challenges Since the Russian Invasion of Ukraine

During the Russian occupation, the mean (SD) staff who left their jobs per hospital was 7.9 (6.4) physicians, 10.3 (9.6) nurses, and 21.6 (18.8) other hospital staff. Although numerous hospital staff left after February 23, 2022, hospitals hired a mean (SD) of 8.3 (7.8) physicians, 10.6 (9.2) nurses, and 26.2 (24.8) other staff members during the Russian occupation, representing a net gain in hospital staff during the war (Figure 1). Relatively small numbers of physicians, nurses, and other hospital staff were injured and died because of the Russian invasion (Figure 1).

**Figure 2.**
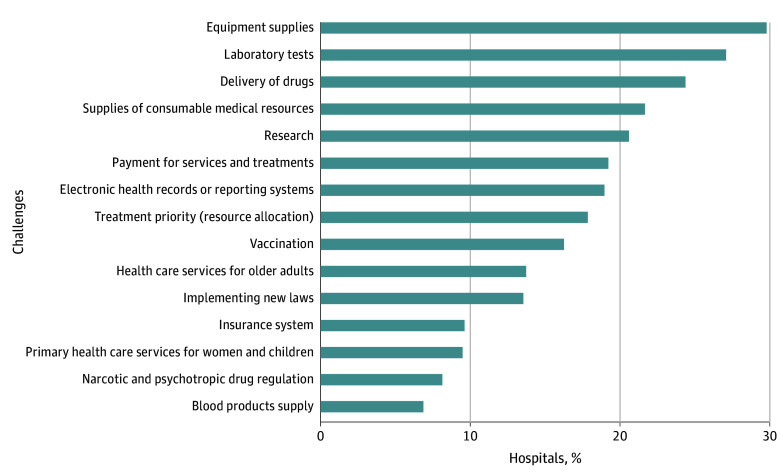
Challenges Faced by the Ukrainian Hospital System During the Russian Invasion

Of the 74 participating hospitals, common problems during the war included 22 hospitals (29.7%) with challenges obtaining supplies, 20 (27.0%) with laboratory test kit shortages, 18 (24.3%) with issues with the delivery of drugs, and 16 (21.6%) with lack of consumable medical resources, such as bandages, needles, and antibiotics (Figure 2).

### Power Outages

**Figure 3.**
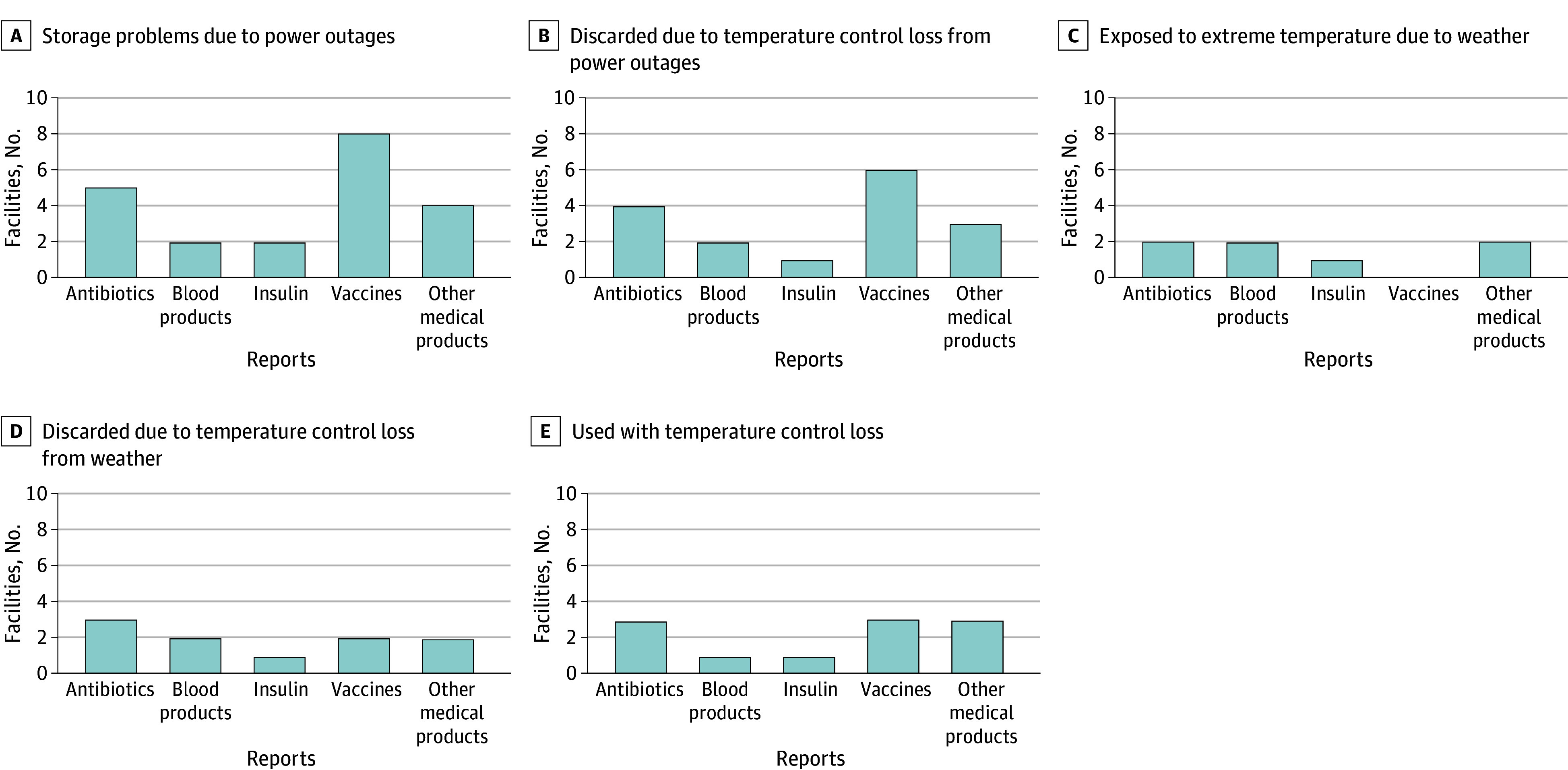
Situations Experienced by Hospitals Since the Russian Invasion of Ukraine, February 24, 2022 These data reflect confirmed reports only. Across measures, 5% to 10% of hospitals lacked a mechanism to verify whether these situations had occurred. Additionally, some measures did not apply to all hospitals based on variations in services provided.

Hospitals reported the outcomes of power outages and winter on the storage and use of 5 types of medical products: antibiotics, blood products, insulin, vaccines, and other medical products. Among the responses, power outages caused more storage problems (eg, vaccines and antibiotics) (Figure 3A), and products were discarded due to loss of temperature control (eg, vaccines and antibiotics) (Figure 3B). Medical products were also exposed to extreme temperatures (eg, antibiotics, blood) (Figure 3C), and medical products were discarded due to loss of temperature control (eg, antibiotics, blood, and vaccines) (Figure 3D). Some hospitals reported the use of antibiotics, vaccines, and other medical products even after loss of temperature control (Figure 3E).

### Overall Changes

The mean (SD) number of physicians and nurses employed by individual hospitals was stable from before to during the war although the number of other staff slightly decreased from 175 (99.5) before the war to 162 (94.7) during the war (eAppendix in Supplement 1). The number of weekly hours worked from before to during the war varied by occupational role, with large increases observed for nurses and other staff. The results of the study further indicated that increasing staff strength (ie, the number of physicians, nurses, and other staff) was significantly associated with job hours increasing from before the war to during the war (eTable 3 in Supplement 1). Resources such as vehicles, ambulances, defibrillators, ventilators, ICU beds, and beds in hospitals significantly decreased from before the invasion to after the invasion (eTable 3 in Supplement 1). In the context of services provided in health care, nonobstetric operating room procedures decreased, while the number of operating rooms for surgical procedures increased. From before to during the war, daily inpatient services (3902 vs 3967) and emergency admissions (2773 vs 2830) both increased (eFigure in Supplement 1). In contrast, from before to during the war, daily outpatient services (10 853 vs 9769) and elective admissions (3127 vs 3422) decreased. Hospital admissions were significantly associated with the availability of hospital resources (ie, beds, ventilators, and ICU beds) and rooms (eTable 3 in Supplement 1). Hospital-reported maternal deaths decreased from 7 before the war to 1 during the war (Table), and deaths for children younger than 5 years were the same from before the war to during the war.

### Attacks on Health Facilities

**Figure 4.**
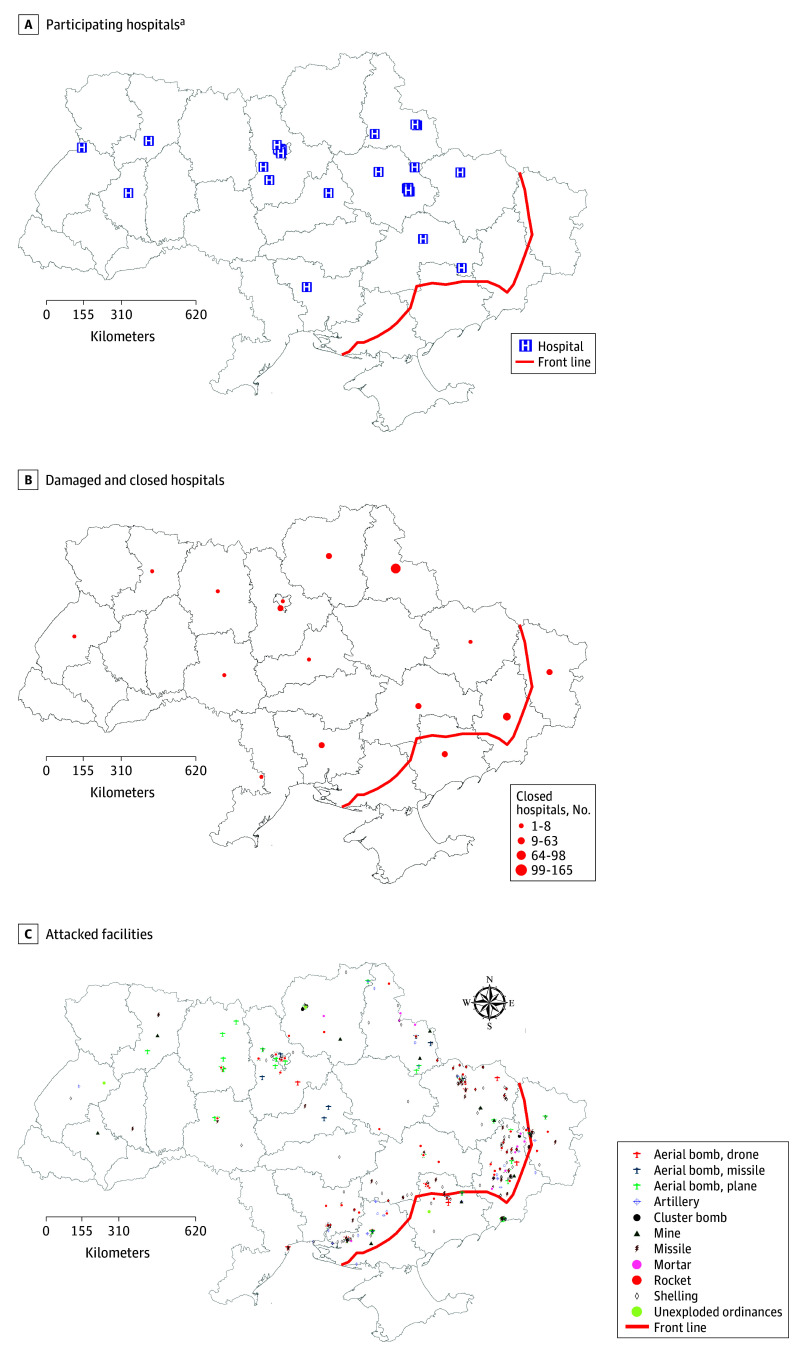
Participating Hospitals, Damage and Closures, and Attacks on Health Facilities in Ukraine ^a^Due to the proximity of multiple hospitals in the same small geographic region, participating hospital icons may be superimposed.

A total of 74 hospitals participated in this study (Figure 4A). Damage to hospitals and health facilities was most common in oblasts along the front lines or adjacent to Russian borders (Figure 4B). Hospitals and health facilities were attacked with artillery, aerial bombs (ie, drone attack or attack from a plane), cluster bombs, mines, missiles, rockets, and shelling (Figure 4C).

## Discussion

During the last 2 years of full-scale war in Ukraine, the largest damage to Ukrainian medical infrastructure has been observed in Kharkiv, Donetsk, Dnipropetrovsk, Mykolaiv, Kherson, Zaporizhzhia, Kyiv, and Chernihiv regions.^16^ In this cross-sectional survey of Ukrainian hospitals, we observed substantial disruptions to hospital services, including reductions in laboratory tests, educational programs, cancer screening, gynecological services, rehabilitation services, reduction in staff numbers, as well as increased job hours for hospital staff. Maternal and newborn health was negatively affected. Substantial turnover in hospital staff and decreases in the availability of essential resources such as vehicles, ambulances, defibrillators, ventilators, ICU beds, and hospital beds during the war were reported. Shifts in the array of services provided were reported, including changes in the volume of nonobstetric operating room procedures, general surgery department operations, and inpatient and outpatient admissions.

These changes in services suggest a shift in hospital priorities and the allocation of resources during the war. General surgical procedures in operating rooms increased during wartime compared with the prewar period.

Not surprisingly, daily inpatient and emergency admissions significantly increased during the war indicating an influx of patients requiring immediate care. In contrast, daily outpatient and elective admissions significantly decreased, likely due to disruption of nonurgent hospital services such as immunization services and interventions to control communicable diseases.

Data from the period of war indicate a disruption in the provision of essential obstetric and neonatal services, likely associated with the migration of medical professionals, the destruction of health facilities, and the displacement of populations. These factors pose a severe risk to the health outcomes of mothers and newborns. Although the maternal deaths and low birth weight cases reported by participating hospitals were almost the same before and during the war, the number of obstetric deliveries at these hospitals also sharply declined, suggesting increases in the rates of these adverse outcomes. These findings are also consistent with other published studies that showed there were increased adverse pregnancy-related outcomes among refugee mothers, including a higher rate of infants with low birth weight, premature births, and miscarriage during this time.^17^ Ensuring continuity of care for pregnant patients, access to safe deliveries, and postnatal care must remain a priority. Mortality rates reported by participating hospitals showed children younger than 5 years and mothers remained the same. It might be because of mass migration; many pregnant and childbearing people have migrated to other countries in search of safe havens. However, the ongoing war may exacerbate by severely disrupting some essential services such as antenatal, childbirth, and postnatal care, including the provision of necessary cesarean deliveries, which, in turn, elevate the risk of life-threatening obstetric and neonatal complications.

During the war, the turnover of health professionals was considerable at the hospitals participating in the survey. The most substantial dropout was observed among staff members. In some areas, hospitals adjusted to staff shortages by engaging community health workers and trained community workers, particularly in areas of increased need (immunization, testing and screening for communicable and chronic diseases, mental health and psychosocial support, and referral and linkage to care).^18^ On average, staffing decreased slightly in hospital facilities during the war compared with before the war, particularly among nurses and other staff. We additionally observed notable increases in job hours among nurses and other staff, which could be due to increased patient care demands as well as the need to compensate for staff shortages. Another challenge during the war was the disruption of hospital supplies.

The Ukrainian energy infrastructure was a major target of Russian attacks, and as a result, many hospitals experienced power outages, and inadequate storage of supplies such as vaccines, antibiotics, drugs, blood, and insulin at the required temperature. Some hospitals reported the need to discard products due to loss of temperature control from power outages, while others reported the use of these products despite known losses of temperature control. Using these products may have negative consequences on human health.

Hospital resources such as vehicles, ambulances, defibrillators, ventilators, ICU beds, and general hospital beds decreased in hospitals during the war compared with before the war. The reason might be some of the hospital assets were bombed.^19^ This reduction in resources may have posed challenges in providing essential medical care and responding to emergencies.

The results suggest a need for continued monitoring of health services among Ukrainian hospitals. For instance, the findings of reductions in access to vaccines may have implications for public health and in particular, infectious disease risk. Vaccination coverage was already low in Ukraine before the war with outbreaks of measles and poliovirus resulting in more than 115 000 measles and 40 deaths from 2017 to 2020.^18,20^ The observed decline in access to vaccines may further exacerbate outbreaks of vaccine-preventable diseases.^21^

Despite the potentially enormous effects on public health, to date, there have been few studies examining changes in hospital services during wartime or military invasions. The approach developed here may lend itself to future investigations in Ukraine as the war continues as well as in other crisis settings where health care systems may be disrupted. The war in Ukraine is the first conflict in which the World Health Organization’s new mandate to document attacks on health care facilities and health care personnel is operating. This is currently the only conflict where the World Health Organization is required to document and confirm these attacks, and this has been the result of years of advocacy and a resolution passed by the World Medical Association.

### Strengths and Limitations

Understanding the outcomes of the war on health services in Ukraine is particularly salient given growing evidence of widespread, deliberate attacks on health care facilities in violation of the rules of war.^11,22^ Planned attacks on health care facilities can be seen as deliberate campaigns to inflict suffering on civilian populations and are considered war crimes subject to adjudication by the International Criminal Court.^23^ Quantifying the potential outcomes of the war on hospital services provides critical data on the humanitarian toll of this ongoing crisis.

We note several limitations of this analysis. Given the challenges of accessing certain regions during the wartime setting, there is a possibility of underrepresentation of heavily affected areas in the data, as well as a potential bias toward regions more accessible during the conflict. Additionally, the inability to include hospitals that were destroyed or rendered inoperable may further limit the representativeness of the findings, potentially leading to an underestimation of the true outcomes of the war on health services in Ukraine. Such factors could result in either overestimation or underestimation of the resilience and adaptability of the hospital system. Findings may be lower bound in that they may not have described changes at hospitals that were destroyed during the war. Additionally, we cannot evaluate how representative the sample of participating hospitals is of all Ukrainian hospitals due to a lack of data on nonparticipating hospitals. The findings presented may be an underestimate of the true outcomes of the war on health services in Ukraine given that hospitals that were destroyed or incapacitated during the war could not respond to the survey. The relatively low response rate may be evidence of this concern. Furthermore, the cross-sectional nature of this study may not fully capture the dynamic changes in health services delivery throughout the war, which is ongoing at the time of acceptance of this article.

## Conclusions

This cross-sectional survey indicates that the Russian invasion of Ukraine has disrupted health services in numerous ways, causing damage to infrastructure and turnover of staff. A decline in preventive services, such as vaccination, and elective services can potentially worsen the health of the population, which will also be adversely affected by exposure to cold temperatures, use of non–temperature-controlled supplies by hospitals, undernutrition, disrupted sleep, and other stressors. With international support, a well-considered and prioritized response is needed to rebuild a smarter health care system that can address new needs while restoring and catching up on essential services that have been lost due to the war. Priorities for public health services in the short to medium term can be to ensure appropriate surveillance, preparedness, and responses to communicable disease threats or outbreaks, expected as a result of lapses in preventive care.

## Notes

Supplement 1. **eAppendix.** Supplemental text **eFigure.** Relationships between factors at two time points (before the war [T1] and during the war [T2]) **eTable 1.** Demographic information of health facilities center **eTable 2.** Change in the availability of healthcare services at Ukrainian hospitals **eTable 3.** Repeated measure ANOVA to evaluate the changes before and during the war in hospitals (n = 74) and relationships between factors at time point T_1_ (before the war) and T_2_ (during the war)

Supplement 2.
Data Sharing Statement

## References

[1] Gesesew H, Berhane K, Siraj ES, . The impact of war on the health system of the Tigray region in Ethiopia: an assessment. BMJ Glob Health. 2021;6(11):e007328. doi:10.1136/bmjgh-2021-007328 34815244 PMC8611430

[2] Taylor L. “Countless lives” at risk in Afghanistan as health services collapse, warns WHO. BMJ. 2023;382:1961. doi:10.1136/bmj.p1961 37620016

[3] Bielka K, Kuchyn I, Horoshko V. Intensive care units during the Ukraine war: challenges and opportunities. Intensive Care Med. 2023;49(8):1011-1014. doi:10.1007/s00134-023-07117-5 37314459

[4] Lafta RK, Al-Nuaimi MA. War or health: a four-decade armed conflict in Iraq. Med Confl Surviv. 2019;35(3):209-226. doi:10.1080/13623699.2019.1670431 31597450

[5] Afzal MH, Jafar AJN. A scoping review of the wider and long-term impacts of attacks on healthcare in conflict zones. Med Confl Surviv. 2019;35(1):43-64. doi:10.1080/13623699.2019.1589687 30943776

[6] Dalton MK, Jarman MP, Manful A, . The hidden costs of war: healthcare utilization among individuals sustaining combat-related trauma (2007-2018). Ann Surg. 2023;277(1):159-164. doi:10.1097/SLA.0000000000004844 33651722

[7] Salmiya MA. Urgent humanitarian call to save lives in Gaza. Lancet. 2023;402(10412):1523-1524. doi:10.1016/S0140-6736(23)02333-4 37865108

[8] Ledur J, Mellen R, Karklis L, Ilyushina M. Wetlands and radioactive soil: how Ukraine’s geography could influence a Russian invasion. 2022. Accessed April 19, 2024. https://www.washingtonpost.com/world/interactive/2022/ukraine-russia-invasion-geography-weather/

[9] Romaniuk P, Semigina T. Ukrainian health care system and its chances for successful transition from Soviet legacies. Global Health. 2018;14(1):116. doi:10.1186/s12992-018-0439-5 30470237 PMC6260664

[10] Dzhus M, Golovach I. Impact of Ukrainian-Russian War on health care and humanitarian crisis. Disaster Med Public Health Prep. 2022;17:e340. doi:10.1017/dmp.2022.265 36474326

[11] The Lancet. Russia’s invasion of Ukraine: an attack on health. Lancet. 2023;401(10377):617. doi:10.1016/S0140-6736(23)00387-2 36841605

[12] 2016-2024 Ukraine attacks on health care incident data. Humanitarian Data Exchange. 2024. Accessed April 19, 2024. https://data.humdata.org/dataset/sind-safeguarding-healthcare-monthly-news-briefs-dataset/resource/2b499763-49f0-4571-90c9-c093934456a0

[13] Haque U, Naeem A, Wang S, . The human toll and humanitarian crisis of the Russia-Ukraine war: the first 162 days. BMJ Glob Health. 2022;7(9):e009550. doi:10.1136/bmjgh-2022-009550 36167408 PMC9511605

[14] Fanaei S, Zareiyan A, Shahraki S, Mirzaei A. Determining the key performance indicators of human resource management of military hospital managers; a TOPSIS study. BMC Prim Care. 2023;24(1):47. doi:10.1186/s12875-023-02007-7 36788481 PMC9926442

[15] Ruangsomboon O, Surabenjawongse U, Jantataeme P, Chawaruechai T, Wangtawesap K, Chakorn T. Association between cardiopulmonary resuscitation audit results with in-situ simulation and in-hospital cardiac arrest outcomes and key performance indicators. BMC Cardiovasc Disord. 2023;23(1):299. doi:10.1186/s12872-023-03320-w 37312018 PMC10265752

[16] Ministry of Health, Ukraine. Over 23 months of war in Ukraine, 480 healthcare facilities have been fully restored and another 372 have been partially restored. Accessed March 3, 2024. https://www.kmu.gov.ua/en/news/za-23-misiatsi-viiny-v-ukraini-povnistiu-vidnovleno-480-obiektiv-medzakladiv-ta-shche-372-obiekty-vidnovleni-chastkovo

[17] Keygnaert I, Ivanova O, WHO Health Evidence Network Synthesis Reports, . What Is the Evidence on the Reduction of Inequalities in Accessibility and Quality of Maternal Health Care Delivery for Migrants? A Review of the Existing Evidence in the WHO European Region. World Health Organization; 2016. https://www.ncbi.nlm.nih.gov/books/NBK390809/27786434

[18] Health Cluster Ukraine. Ukraine public health situation analysis (PHSA)–short-form. Accessed March 3, 2023. https://reliefweb.int/sites/reliefweb.int/files/resources/ukraine-phsa-shortform-030322.pdf

[19] World Health Organization. WHO records more than 1000 attacks on health care in Ukraine over the past 15 months of full-scale war. May 30, 2023. Accessed on December 19, 2023. https://www.who.int/europe/news/item/30-05-2023-who-records-1-000th-attack-on-health-care-in-ukraine-over-the-past-15-months-of-full-scale-war

[20] Hill M, Vanderslott S, Volokha A, Pollard AJ. Addressing vaccine inequities among Ukrainian refugees. Lancet Infect Dis. 2022;22(7):935-936. doi:10.1016/S1473-3099(22)00366-8 35752178 PMC9221088

[21] Orsini D, Martini M. Measles: a new danger for Ukraine’s children—the need for an effective and timely vaccination prevention campaign for an insidious disease that comes from afar. J Prev Med Hyg. 2023;64(2):E204-E208. 37654850 10.15167/2421-4248/jpmh2023.64.2.2996 PMC10468189

[22] Global Centre for the Responsibility to Protect. Resolution 2286 (Protection of Civilians) S/RES/2286. May 3, 2016. https://www.globalr2p.org/resources/resolution-2286-protection-of-civilians-s-res-2286/

[23] Dormann K. War crimes under the Rome Statute of the International Criminal Court, with a special focus on the negotiations on the elements of crimes. In: von Bogdandy A, Wolfrum R, eds. Max Planck Yearbook of United Nations Law. Vol 7. Martinus Nijhoff Publishers; 2003. doi:10.1163/187574103X00077

