# Error in Author Name

**DOI:** 10.1001/jamahealthforum.2025.5794

**Published:** 2025-11-14

In the Original Investigation titled “A Comparison of Ukrainian Hospital Services and Functions Before and During the Russia-Ukraine War,”^1^ published online on May 17, 2024, the name of the 13th author was misspelled. “Ifthekar Sikder” should be “Iftikhar U. Sikder.” This article was corrected online.
