# Supplementary material for: A Comparison of Ukrainian Hospital Services and Functions Before and During the Russia-Ukraine War
Source: JAMA Health Forum. 2024 May 17;5(5):e240901. doi: 10.1001/jamahealthforum.2024.0901 (PMC11102023; doi:10.1001/jamahealthforum.2024.0901)
Supplement: Supplement 1. — eAppendix. Supplemental text eFigure. Relationships between factors at two time points (before the war [T1] and during the war [T2]) eTable 1. Demographic information of health facilities center eTable 2. Change in the availability of healthcare services at Ukrainian hospitals eTable 3. Repeated measure ANOVA to evaluate the changes before and during the war in hospitals (n = 74) and relationships between factors at time point T1 (before the war) and T2 (during the war) [file jamahealthforum-e240901-s001.pdf]

Supplemental Online Content

Haque U, Bukhari MH, Fielder N, et al. A comparison of Ukrainian hospital services and functions before and during the Russia-Ukraine War. *JAMA Health Forum*. 2024;5(5):e240901. doi:10.1001/jamahealthforum.2024.0901

eAppendix. Supplemental text

eFigure. Relationships between factors at two time points (before the war [T1] and during the war [T2])

eTable 1. Demographic information of health facilities center

eTable 2. Change in the availability of healthcare services at Ukrainian hospitals

eTable 3. Repeated measure ANOVA to evaluate the changes before and during the war in hospitals (n = 74) and relationships between factors at time point T_1 (before the war) and T_2 (during the war)

This supplemental material has been provided by the authors to give readers additional information about their work.

© 2024 Haque U et al. *JAMA Health Forum.*

eAppendix. Supplemental text

Hospital Facility Level

National-level hospital: National-level hospitals in Ukraine are typically high-profile medical institutions that provide specialized and advanced medical services. They are often located in major cities and serve as referral centers for complex medical cases.

Oblast-level hospital: Oblast-level hospitals are regional or provincial hospitals that serve a specific administrative region known as an "oblast" in Ukraine. These hospitals provide a comprehensive range of medical services and are referral points for primary and secondary care facilities within the respective oblast.

City-level hospital: City-level hospitals serve the population of a particular city. They vary in size and scope of services, with some offering specialized care in addition to general medical services. City-level hospitals are an important part of the healthcare infrastructure and cater to the healthcare needs of urban residents.

District-level hospital: District-level hospitals are located at the district level and provide healthcare services to the population of a specific administrative district or raion in Ukraine. They primarily offer primary and secondary care services, including general medical care, basic surgeries, and emergency care. These hospitals serve as the first point of contact for many patients and may refer more complex cases to higher-level facilities.

Form of Ownership

State hospitals: State hospitals in Ukraine are owned, funded, and operated by the government, typically at the national or regional level. These hospitals are part of the public healthcare system and provide medical services to the population.

© 2024 Haque U et al. *JAMA Health Forum.*

Communal hospital: Communal hospitals in Ukraine are owned and operated by local communities or municipalities. They are funded through local government budgets and may serve specific cities, towns, or districts.

Private hospitals: Private hospitals in Ukraine are owned and operated by private individuals, organizations, or companies. These hospitals are privately funded and managed, and they may offer a wide spectrum of medical services.

Best Facility Centers

Primary healthcare: Primary healthcare in Ukraine serves as the first point of contact between individuals and the healthcare system. It focuses on delivering essential, preventive, and basic medical services. Primary healthcare providers, including family doctors and general practitioners, offer services such as health promotion, disease prevention, diagnosis and treatment of common health issues, and referrals to higher-level healthcare facilities when needed.

Secondary healthcare level: Secondary healthcare facilities in Ukraine are equipped to provide more specialized medical services than primary care facilities. These facilities often include district hospitals, regional hospitals, and specialty clinics. They offer a broader range of diagnostic and treatment services, including surgeries, specialized medical consultations, and emergency care.

Tertiary healthcare level: Tertiary healthcare facilities in Ukraine are typically large, specialized medical centers that offer advanced and highly specialized medical services. These hospitals may include national or regional referral hospitals, teaching hospitals, and medical research institutions. They provide complex medical treatments, specialized surgeries, and care for severe and rare medical conditions.

Palliative care: Palliative care in Ukraine focuses on providing holistic and compassionate care for individuals with life-limiting illnesses. The goal of palliative care is to enhance the quality of life, alleviate pain and suffering, and provide emotional and psychological support to patients and their families. Palliative care may be offered in various settings, including hospices, home care, and inpatient

© 2024 Haque U et al. *JAMA Health Forum.*

facilities. It addresses the physical, emotional, social, and spiritual needs of patients and is provided by a multidisciplinary team.

Type of Hospital

Regional hospital (adults and children): A regional hospital in Ukraine is a medical facility that serves a specific administrative region or oblast. Regional hospitals typically provide a wide range of medical services to both adults and children. These hospitals offer general medical care, and surgical services, and may have specialized departments for various medical specialties.

Infectious regional hospital: An infectious regional hospital is a specialized healthcare facility that focuses on the diagnosis, treatment, and prevention of infectious diseases. These hospitals are equipped to manage patients with contagious illnesses to prevent the spread of infections to the general population. They often have isolation units and specialized medical staff.

Tuberculosis regional hospital: A tuberculosis regional hospital is a specialized facility dedicated to the diagnosis, treatment, and care of patients with tuberculosis (TB). TB hospitals play a crucial role in managing TB cases, providing appropriate treatment regimens, and ensuring infection control to prevent the spread of the disease.

Town Hospital: A town hospital in Ukraine serves the healthcare needs of the population within a specific town or city. Town hospitals offer general medical services, and emergency care, and may have basic surgical capabilities.

Central District Hospital: A central district hospital is a healthcare facility serving a particular administrative district or raion within Ukraine. A raion often translated as district, is the second-level administrative division in Ukraine. Central district hospitals offer a range of healthcare services, including primary care, and general medical services, and may provide limited specialized care. They serve as the primary point of contact for many patients in the district and may refer more complex cases to higher-level facilities.

© 2024 Haque U et al. *JAMA Health Forum.*

eFigure. Relationships between factors at two time points (before the war [T1] and during the war [T2])

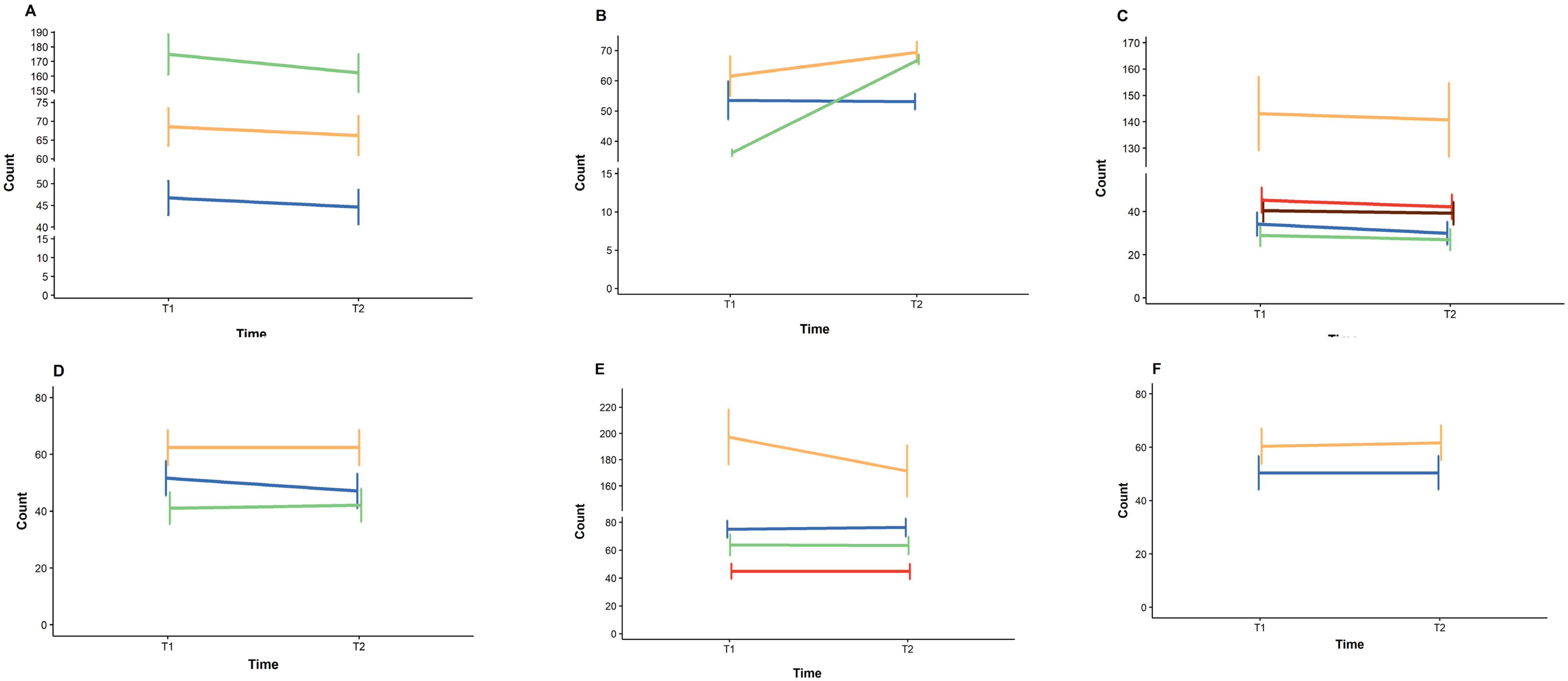

Figure S1: A: Number of hospital Staff (doctor, nurse, other staff), Blue color represents: Doctor, Orange: Nurse, and Green: Other Staff. P-value<0.0001, F statistics: 115.801, B: Hospital Staff weekly total hours (doctor, nurse, other staff), Blue color represents Doctors, Orange: nurses, and Green: other staff. P-value:0.0003., F statistics: 8.281, C: Ambulances, Hospital beds, ICU beds, ventilators, defibrillation, Green color represents: defibrillation, blue: ambulances, brown: ventilators, Red: ICU beds, Orange: Hospital Beds, F statistic:70.275; p-value: <0.0001, D: Non-obstetrical Operating rooms procedures, operating rooms to procedures hospital facility perform, obstetric deliveries hospital facility performs, Green color represents operating rooms to procedures hospital facility perform, Orange: obstetric deliveries, Blue: non-obstetrical Operating rooms procedures. P-value:0.003, F statistics: 6.03, E: Average daily emergency admissions, elective admissions, inpatients, outpatients, Red: Average daily emergency admissions, Green: elective admissions, Blue: inpatients, and Orange: outpatients, P-value:<0.0001, F statistics: 52.189, F: Monthly average maternal deaths, and under-five years (age) of deaths, Blue color represent under-five years (age) of deaths, Orange: Monthly average maternal deaths, P-value: 0.065, F statistics: 3.488.

© 2024 Haque U et al. JAMA Health Forum.

eTable 1. Demographic Information of Health Facilities Center

		Frequency (%)
**Hospital facility level**	National level	7 (9.46)
	Oblast level	16 (21.62)
	City level	39 (52.70)
	District level	12 (16.22)
**Form of ownership**	State	08 (10.81)
	Communal	60 (81.08)
	Private	06 (8.11)
**Best facility centers**	Primary hospital & Preventive	18 (24.32)
	Secondary hospital level	39 (52.70)
	Tertiary hospital level	16 (21.62)
	Palliative care	1 (1.35)
**Type of hospital**	Regional Hospital (adults and children)	23 (31.08)
	Infectious Regional Hospital	1 (1.35)
	Tuberculosis Regional Hospital	3 (4.05)
	Town Hospital	15 (20.27)
	Central District Hospital	16 (21.62)
	Others	16 (21.62)
**Oblast/ State**	Dnipropetrovsk	2 (2.70%)
	Kharkiv	41 (55.41%)
	Kyiv	11 (14.86%)
	Kirovohrad	1 (1.35%)
	Lviv	1 (1.35%)
	Mykolaiv	1 (1.35%)
	Poltava	9 (12.16%)
	Rivne	2 (2.70%)
	Sumy	5 (6.76%)
	Ternopil	1 (1.35%)

© 2024 Haque U et al. *JAMA Health Forum.*

eTable 2. Change in the availability of healthcare services at Ukrainian hospitals

Healthcare Services	Before the war n (%)	During the war n (%)	% reduction
≥ 25% decrease in number of hospitals offering service			
Monkeypox vaccines	2(3)	1(1)	50%
Telehealth facilities	33(45)	21(28)	36%
Oral health	28(38)	19(26)	32%
Educational Program for Tobacco Use	52(70)	36(49)	31%
Blood Donor Center	17(23)	12(16)	29%
Rehabilitation services	37(50)	27(36)	27%
Emergency dental services	26(35)	19(26)	27%
Blood Bank	19(26)	14(19)	26%
Gynecological services	43(58.)	32(43)	26%
Nursing home	4(5)	3(4)	25%
Pharmacy	36(49)	27(36)	25%
10-24% decrease in number of hospitals offering service			
Cancer Screening	49(66)	37(50)	24%
Emergency department	14(19)	11(15)	21%
Orthopedic services	34(46)	27(36)	21%
Urgent care	50(68)	43(58)	14%
Routine pediatric services	38(51)	33(45)	13%
Sexual abuse victims resources	8(11)	7(9)	13%
Emergency abortion care	16(22)	14(19)	13%
Ambulatory surgery center	32(43)	28(38)	13%
Mental health services	32(43)	28(38)	13%
Laboratory tests	72(97)	63(85)	13%
COVID-19 vaccines	43(58)	38(51)	12%
General Surgery Department	36(49)	32(43)	11%
<10% decrease in number of hospitals offering service			
Routine prenatal care	25(34)	23(31)	8%
Imaging and Radiology Center	55(74)	51(69)	7%
Intensive care unit	44(59)	42(57.)	5%
Vaccinations for children	31(42)	30(41)	3%
Dialysis Center	5(7)	5(7)	0%
Addiction treatment center	5(7)	5(7)	0%
Obstetric deliveries	11(15)	11(15)	0%

© 2024 Haque U et al. *JAMA Health Forum.*

eTable 3. Repeated Measure ANOVA to evaluate the changes before and during the war in hospitals (n=74) and relationships between factors at time point T1 (before the war) and T2 (during the war)

**Outcome Variables**	Average		*F-statistic*	*p-value*
	Pre-war	*During-war*		
Staff Strength	95.34	88.94	115.801	*<0.0001*
Staff Job Hours	49.6	63.4	8.281	0.0003
Staff Role (strength) x Staff Job Hours	-	-	52.476	<0.0001
Hospital Resources	58.0	55.4	70.275	*<0.0001*
Hospital Rooms	51.3	50.2	6.03	0.003
Patient Admissions	88.70	94.58	52.189	<0.0001
Hospital Furniture **x** Patient Admissions	-	-	6.649	0.01
Hospital Rooms **x** Patient Admissions	-	-	9.985	0.002
Patient Deaths	55	56.2	3.488	0.065

© 2024 Haque U et al. JAMA Health Forum.
