# Supplementary material for: A Comparison of Ukrainian Hospital Services and Functions Before and During the Russia-Ukraine War
Source: JAMA Health Forum. 2024 May 17;5(5):e240901. doi: 10.1001/jamahealthforum.2024.0901 (PMC11102023; doi:10.1001/jamahealthforum.2024.0901)
Supplement: Supplement 2. — Data Sharing Statement [file jamahealthforum-e240901-s002.pdf]

# Data Sharing Statement

Haque. A Comparison of Ukrainian Hospital Services and Functions Before and During the Russia-Ukraine War. *JAMA Health Forum*. Published May 17, 2024.
doi:10.1001/jamahealthforum.2024.0901

**Data**
**Data available:** Yes
**Data types:** Deidentified participant data, Data (not involving human participants)
**How to access data:** Data will be shared based on request
**When available:** With publication

**Supporting Documents**
**Document types:** None

**Additional Information**
**Who can access the data:** Researchers
**Types of analyses:** NA
**Mechanisms of data availability:** with a signed data access agreement
